# Sleep Quality and Emotional State of Medical Students in Dubai

**DOI:** 10.1155/2022/8187547

**Published:** 2022-02-14

**Authors:** Hamdah Meer, Lakshmanan Jeyaseelan, Meshal A. Sultan

**Affiliations:** ^1^Dubai Medical College for Girls, Dubai, UAE; ^2^College of Medicine, Mohammed Bin Rashid University of Medicine and Health Sciences, Dubai, UAE; ^3^Mental Health Centre of Excellence, Al Jalila Children's Speciality Hospital, Dubai, UAE

## Abstract

Poor sleep quality has been reported to be common amongst medical students and healthcare professionals worldwide. Sleep disturbance has been associated with increased rates of burnout and depression. As a result, this has been negatively impacting performance and functioning. Research on this topic is limited in the United Arab Emirates (UAE). This study is aimed at exploring sleep pattern and the emotional state of medical and dental students in Dubai, UAE. This cross-sectional study was based on an electronic survey sent to 181 medical and dental students. Of the 181 invitations, 96 individuals agreed to participate and complete the rating scales. The Pittsburgh Sleep Quality Index was utilized to explore sleep duration, quality, and daytime functioning. The Positive and Negative Affect Scale was used to assess the emotional states of the participants. Overall, the findings revealed diminished sleep duration. The average duration of sleep amongst the study participants was 5 hours and 24 minutes, which is significantly below the recommended duration as per sleep guidelines. Results also showed a significant positive correlation between total sleep duration and overall sleep quality with enthusiasm during the day. Future research designed to explore factors contributing to sleep efficiency, in more depth, as well as strategies to enhance sleep quality is highly warranted.

## 1. Introduction

Mental illness refers to a wide range of mental health conditions that affect an individual's mood, thinking, and behaviour [1]. Adopting good sleep hygiene results in enhanced physical and psychological wellbeing, e.g., improved cardiovascular function, metabolism, immune system, attention, cognition, memory, and mood regulation [2]. Research has shown that lack of sleep was associated with a significant adverse impact in performance in exams [3]. Furthermore, studies have shown high rates of inadequate sleep amongst medical students [4].

“Poor sleep quality was present in 30%, excessive daytime sleepiness (EDS) in 40%, and insomnia symptoms in 33% of students” according to a study conducted on 305 medical students [5]. Other studies conducted on medical students and on youth report a significant presence of emotional exhaustion and significant reduction in sleep quality in the majority of the participants [6, 7].

Research exploring the effect of sleep on psychological wellbeing in the United Arab Emirates (UAE) has been very limited. This study is aimed at answering the following question: “Is there an association between sleep quality and the emotional state of medical and dental students at Mohamed Bin Rashid University of Medicine and Health Sciences in the year 2019?” The findings aim to provide recommendations for enhancing sleep quality and education on its fundamental role in overall wellbeing.

## 2. Methods

### 2.1. Participants and Procedures

This study is an observational analytical cross-sectional study. The participants were medical and dental students enrolled at Mohamed Bin Rashid University of Medicine and Health Sciences (MBRU) in the year 2019. The study took place in Dubai, United Arab Emirates (UAE). The duration of the study was 10 months, where data collection was conducted from May to August 2019. The final data analysis was concluded by the end of September 2019. This study was reported according to the standards of the STROBE guidelines [8].

**Figure 1:**
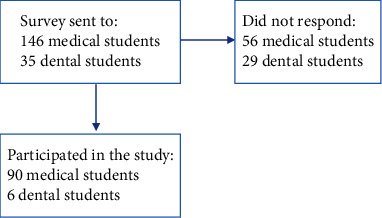
Flow chart of participants.

The criteria of inclusion in this study are both medical and dental students enrolled at MBRU in the year 2019. There were no exclusion criteria used in the study during the recruitment of MBRU medical and dental students. All the students at MBRU were given the opportunity to take part in the study by completing an online survey that was sent by email. No sample size was statistically calculated because of the technique in obtaining the coverage (complete coverage). The finalized sample size is based on the number of students that completed the survey. Students were recruited from year one, two, and three from the college of medicine as well as dental students in the postgraduate program. MBRU has been inaugurated in 2016, and all batches were included in this study (Figure 1).

### 2.2. Measurements

Several variables were assessed in the study classified into three groups, demographic data, sleep quality, and emotional state. The demographic data included age, gender, nationality, and medical/dental student. The self-rating tools used were the Pittsburgh Sleep Quality Index (PSQI) [9] and the Positive and Negative Affect Scale (PANAS) [10].

The PSQI evaluated sleep parameters over the past month. The variables included time to go to bed at night, time to fall asleep, time to wake up in the morning, and the total duration of sleep. The PSQI also included variables related to sleep difficulty, including frequency of not being able to sleep within 30 minutes, waking up in the middle of the night or early morning, getting up to use the bathroom, difficulties in breathing comfortably, coughing or snoring loudly, feeling too cold, feeling too hot, having bad dreams, having pain, taking medicine to help with sleep, and trouble staying awake whilst driving, eating meals, or engaging in social activities. The PSQI also evaluated functioning during the day, for example, “difficulty in staying awake during the day” and “keeping up enough enthusiasm to get things done.” Additionally, the PSQI included a subjective rating of “during the past month, how would you rate your sleep quality overall?” The overall sleep quality ranged between the following four options: “Very good,” “Fairly good,” “Fairly bad,” and “Very bad.” The Global PSQI score was calculated for each participant as per the “Scoring Instructions for the Pittsburgh Sleep Quality Index” [9].

The PANAS evaluated different feelings and emotions over the past month. The participants were expected to choose one of the following five options for each variable: “Very little or not at all,” “A little,” “Moderately,” “Quite a bit,” or “Extremely.” The positive feelings and emotions were interested, excited, enthusiastic, strong, proud, inspired, determined, alert, attentive, and active. The negative feelings and emotions were distressed, upset, guilty, ashamed, scared, afraid, nervous, hostile, irritable, and jittery.

The survey was designed by utilizing both scales on Google Form. The form was sent to all the students at MBRU via a link sent by email. The resultant information was extracted into a Microsoft Excel sheet and saved prior to exporting the data for analysis, using a data analysis software.

### 2.3. Statistical Analysis

The estimated study size was 181 students from both medical and dental colleges enrolled at MBRU in the year 2019. Of the 181 students, 96 students participated in the study. The data obtained from the surveys was exported into the Statistical Package of Social Sciences (SPSS) version 24 and analyzed using SPSS [11].

The categorical variables are presented as numbers and percentages, and the continuous variables are presented as means and their standard deviation. The analyses of variance was used as parametric test, and the Kruskal Wallis test as nonparametric test, as appropriately based on the shape of the distribution of continuous variables such as age and time to sleep. The Cronbach's alpha was computed separately for positive emotions and negative emotions. The correlation between the two categorical variables was done using Spearman's rank correlation. The correlation between a continuous and categorical variable was done using Kendall's Tau correlation coefficient. To compare the two group means of emotions in Likert scale, Mann–Whitney *U* test was used. The *p* value less than 5% was considered for statistical significance.

### 2.4. Ethical Approval and Informed Consent

Ethical approval was obtained from the Institutional Review Board (IBR) of MBRU, to proceed with this study (IRB approval number MBRU-IRB-SRP-78-2017). No student was excluded from this study based on race, gender, religious, or cultural backgrounds. Student privacy was maintained by keeping the data collected anonymous and password protected and only accessible by the main researchers in this study. Identifying demographics was not obtained during data collection.

Consent was obtained from all participants aged 18 years and above. Medical students younger than 18 years of age required their parental/guardian consent, along with their consent, before participating. Information regarding the survey was provided at the beginning of the questionnaire by email along with informed consent prior to taking part in the survey. Students had the choice to not answer specific questions in the questionnaire.

## 3. Results

A total of 96 students responded to the questionnaire. Of the participants studied, 22% were males and 77% were females. The mean age (sd) of male participants was 20.1 (2.5) years and 20.0 (3.6) years for female participants. Approximately, 40% of the subjects were Emiratis and 60% were from other countries. The mean age of both groups was approximately 20 years. 94% were from the medical college and only 6% percent were from the dental college. The mean (sd) age of medical students was 19.5 (2.4) years and 29.3 (3.8) years for the dental students. The difference in age amongst students from the two specialties was statistically significant (*p* < 0.001).  Table 1 presents the number, percentage, and mean of age of the study subjects according to sociodemographic variables.

The Global PSQI was calculated for participants with complete data (*n* = 89). Scores greater than 5 points were used to indicate the presence of poor quality of sleep [9]. The mean (sd) Global PSQI score of the study participants was 8.2 points (2.9). The percentage of participants with a score suggestive of poor sleep quality was 84.3.

In terms of reliability for PSQI, the overall Cronbach's alpha was 0.832 (95% CI: 0.77, 0.87), indicating a high degree of internal consistency. In terms of the overall reliability for PANAS, Cronbach's alpha was 0.78 (95% CI: 0.71, 0.84) (*p* = 0.001). For positive emotions, it was 0.90 (95% CI: 0.87, 0.93) (*p* = 0.001). For the negative emotions, it was 0.87 (0.84, 0.91) (*p* = 0.001).

Table 2 presents the overall sleep quality over the past month and sleep time-related variables as per the PSQI in relation to mean age and its standard deviation (SD). The findings indicated that there was no significant difference in age by quality of sleep. However, the subjects who woke up later, at around 9 o'clock, had a very good quality of sleep as compared to others (*p* = 0.047). The subjects who had less hours sleep at around 4-5 hours in total had either very bad or fairly bad quality of sleep as compared to others (*p* < 0.001). Approximately 50% of the participating students reported going to bed at night between 1 : 00 am to 3 : 00 am.

Table 3 demonstrates the mean values and standard deviations (SD) for the reported time to fall asleep as well as total hours of sleeping per night over the past month. On average, it took the participants approximately 25 minutes to fall asleep (SD = 33.5). The mean (sd) total duration of sleep in the study sample was 5.4 (1.6) hours.

Findings based on the Pittsburg Sleep Quality Index (PSQI) revealed high rates of self-reported poor-quality sleep amongst the participants. Furthermore, 43.6% of the total participants had difficulty falling asleep within 30 minutes at least once a week. Additionally, 37.3% of the participants had interrupted sleep at least once weekly (Table 4).

Table 5 shows the correlation between sleep disturbance/use of sleeping medication/daytime functioning with the total duration of sleep and the self-rated overall sleep quality. Frequency of “difficulty in staying awake during the day” was significantly negatively correlated with total duration of sleep and with the overall sleep quality. The correlation coefficients were -0.333 and -0.321 (*p* < 0.001), respectively. However, the variable on “keeping up enough enthusiasm to get things done” was significantly positively correlated with total duration of sleep and with overall sleep quality. The correlation coefficients were 0.384 and 0.388 (*p* < 0.001), respectively.

Table 6 shows the mean and its standard deviation (sd) of the positive and negative emotions by gender. The self-rating for emotions ranged between “1 = Very little or not at all,” “2 = A little,” “3 = Moderately,” “4 = Quite a bit,” and “5 = Extremely.” In general, most of the emotions differed by gender. However, the mean (sd) of the negative emotion “Scared” was 3.1 (1.3) for females as compared to 2.1 (1.2) for males (*p* = 0.001). Similarly, females had significantly higher mean for “Afraid” 3.0 (1.4) as compared to males 1.9 (1.1) (*p* = 0.001), respectively. Similarly, females had significantly higher mean (sd) scores for “Nervous” 3.7 (1.2) as compared to males 2.6 (1.3) (*p* = 0.001).

## 4. Discussion

This study has shown total duration of sleep amongst participants as being below recommended guidelines [12, 13]. The average duration of sleep was 5 hours and 24 minutes (SD = 1 hour and 36 minutes), which is below guidelines recommendation of 8 to 10 hours of nighttime sleep for youth [12, 13]. Various studies have made recommendations for enhancing sleep quality, for instance, adequate sleep duration [14], maintaining regular bedtime and waketime [15], avoiding excessive caffeine intake [16], and limiting screen-time exposure before bed [17, 18].

Previous literature has demonstrated that medical students have poor sleep quality and low quantity [5, 19–22]. These findings are consistent with the results of sleep quality derived in our study. Our study revealed that the average duration of sleep for female students and male students differed, which has also been observed in other studies [23]. Furthermore, our study revealed that anxiety-related emotional states, for instance, “Scared,” “Afraid,” and “Nervous,” were significantly higher amongst females.

Studies have explained the importance of sleep towards various cognitive functions, including memory and emotional regulation [24]. Furthermore, functional neuroimaging studies have shown that individuals experiencing sleep deprivation demonstrate stronger reaction of the amygdala to negative stimuli [24]. A study evaluating sleep loss amongst medical interns reported a negative impact on performance [25]. Moreover, irritability and forgetfulness were reported by the medical interns who worked on consecutive night shifts [25]. In keeping with the abovementioned findings of previous studies, the analysis of the results of our study revealed a positive significant correlation between sleep quality and enthusiasm and productivity during the day. The correlation coefficient of overall sleep quality with enthusiasm was 0.388 (*p* < 0.01).

### 4.1. Strengths and Limitations

One of the strengths of this study is that all medical and dental students at MBRU were invited to participate in the survey. The participants answered almost all the questions in the survey. The participation rate was 53% (96 out of 181 students). The results obtained were consistent with the findings in previous studies. Additionally, this is one of the first studies exploring the relationship between sleep quality and emotional states amongst medical students in Dubai, UAE.

The study's limitations include language bias, since only research papers in English language were reviewed. Moreover, response bias was implicated in this study in the context of the participation rate mentioned above. Other potential biases include selection bias where only students from MBRU were enrolled into the study, as well as recall bias as participants may not be able to accurately remember details related to their sleep and emotional state. Additionally, various confounding factors, which may contribute to sleep quality and psychological well-being, have not been assessed, including life stressors, coping strategies, and presence of medical and mental disorders. Furthermore, additional studies with larger sample sizes are needed to be able to generalize the results of this study.

### 4.2. Implications and Areas for Future Research

This study sheds light on the overall sleep quality and duration as well as emotional states of the participating medical and dental students. In-depth evaluation of contributing factors from a biopsychosocial perspective will assist in better understanding of this subject. This will also assist in designing awareness and educational programs accordingly. Future studies that include a comparison group form the general population can assist in evaluating the extent of sleep challenges amongst medical students. Furthermore, additional multisite studies with larger sample sizes are highly warranted.

## 5. Conclusion

This study illustrated relatively low quality and decreased duration of sleep amongst medical and dental students at MBRU. It also revealed a gender difference in total sleep duration. Additionally, it has shown a significant relationship between adequate sleep and feelings of enthusiasm during the day. The findings highlight the importance of promoting awareness and education on sleep hygiene. Future research assessing this area in more detail will assist in the development of support strategies, which can contribute to improving medical and dental students' overall experience throughout their academic journey.

## Figures and Tables

**Table 1:** The distribution of study subjects by sociodemographic variables and the mean age.

	*N* (%)	Mean age (years)	SD	*p* value
Gender:				
Female	74 (77.1)	20.0	3.6	0.903
Male	21 (21.9)	20.1	2.5	
Nationality:				
Emirati	38 (39.6)	19.7	3.4	0.407
Others	58 (60.4)	20.3	3.5	
Speciality type:				
Medical	90 (93.8)	19.5	2.4	<0.001
Dental	6 (6.3)	29.3	3.8	

*N*: number; %: percentage; SD: standard deviation.

**Table 2:** Overall sleep quality based on age and sleep time-related variables.

	Rate of overall sleep quality								
	Very bad		Fairly bad		Fairly good		Very good		*p* values
	Mean	SD	Mean	SD	Mean	SD	Mean	SD	
Age (years)	20.0	3.5	19.5	3.2	20.7	3.5	22.7	4.5	0.156
Time to fall asleep (minutes)	21.8	25.8	31.1	44.0	19.0	17.2	15.2	13.3	0.403
Time to go to bed at night (o'clock)	5.0	5.4	7.3	7.3	9.7	8.3	12.5	9.5	0.073
Time to wake up in the morning (o'clock)	6.9	1.3	7.1	1.3	7.3	2.5	9.3	3.5	0.047
Total duration of sleep (hours)	4.2	1.4	5.2	1.2	6.2	1.4	7.5	1.0	<0.001

SD: standard deviation.

**Table 3:** Average time to fall asleep and total duration of sleep based on gender.

	Total sample		Gender				
			Female		Male		*p* value
	Mean	SD	Mean	SD	Mean	SD	
Time to fall asleep (minutes)	24.8	33.5	27.1	37.3	17.0	12.3	0.64
Total duration of sleep (hours)	5.4	1.6	5.3	1.6	5.8	1.5	0.25

SD: standard deviation.

**Table 4:** Sleep quality amongst participants based on gender.

		Gender				*p* value
		Female		Male		
		Count	*N* %	Count	*N* %	
Overall sleep quality	Very bad	19	20.0%	2	2.1%	
	Fairly bad	31	32.6%	12	12.6%	0.351
	Fairly good	20	21.1%	5	5.3%	
	Very good	4	4.2%	2	2.1%	
Frequency of difficulty in falling asleep within 30 minutes	Not during the past one month	29	30.9%	10	10.6%	
	Less than once a week	9	9.6%	5	5.3%	0.329
	Once or twice a week	17	18.1%	2	2.1%	
	Twice of three times a week	18	19.1%	4	4.3%	
Frequency of waking up in the middle of the night or early morning	Not during the past one month	28	29.8%	9	9.6%	
	Less than once a week	16	17.0%	6	6.4%	0.399
	Once or twice a week	15	16.0%	1	1.1%	
	Twice of three times a week	14	14.9%	5	5.3%	

**Table 5:** Correlation between sleep disturbance/use of sleeping medication/daytime functioning with the total duration of sleep and the self-rated overall sleep quality.

	Total duration of sleep	Overall sleep quality
Sleep disturbance:		
Frequency of breathing difficulty	-0.218^∗^	0.082
Frequency of coughing or snoring loudly	-0.106	-0.125
Frequency of pain	-0.222^∗^	0.150
Use of sleeping medication:		
Frequency of taking sleeping medication	-0.215^∗^	-0.167
Daytime functioning:		
Frequency of difficulty in staying awake during the day	-0.333^∗∗^	-0.321^∗∗^
Keeping up enough enthusiasm to get things done	0.384^∗∗^	0.388^∗∗^

Correlation coefficients are presented in the above table ^∗^ − *p* < 0.05, ^∗∗^ − *p* < 0.01, and ^∗∗∗^ − *p* < 0.001.

**Table 6:** Mean, standard deviation of positive and negative emotions by gender.

	Male		Female		
	Mean	Standard deviation	Mean	Standard deviation	*p* value
Distressed	3.00	0.95	3.31	1.13	0.189
Upset	2.62	1.02	3.01	1.18	0.189
Ashamed	1.90	1.14	2.08	1.19	0.562
Scared	2.10	1.22	3.11	1.38	0.004
Afraid	1.90	1.14	3.04	1.41	0.001
Nervous	2.57	1.29	3.72	1.24	0.001
Hostile	1.86	0.85	2.39	1.38	0.202
Irritable	3.05	1.02	3.32	1.33	0.305
Jittery	2.48	1.17	2.93	1.38	0.173
Guilty	2.52	1.44	2.84	1.38	0.380
Interested	2.95	1.12	2.66	.92	0.235
Excited	2.67	1.20	2.54	1.01	0.692
Enthusiastic	2.86	1.28	2.51	1.05	0.230
Proud	2.62	1.12	2.57	1.09	0.813
Alert	2.95	1.02	2.73	1.05	0.413
Inspired	2.43	1.03	2.72	1.09	0.304
Determined	3.05	1.07	3.01	1.20	0.830
Attentive	2.57	1.08	2.62	0.99	0.940
Active	3.11	1.24	2.65	1.18	0.139
Strong	2.81	0.87	2.65	0.97	0.390

## Data Availability

The datasets and materials used and/or analyzed during the current study can be made available from the corresponding author on reasonable request.
